# Peak saccadic eye velocity across menstrual phases in naturally cycling women; A pilot study

**DOI:** 10.1016/j.cpnec.2020.100009

**Published:** 2020-09-17

**Authors:** Taran Giddey, Natalie Thomas, Abdul-Rahman Hudaib, Elizabeth H.X. Thomas, Jessica Le, Paige Gray, Caroline Gurvich

**Affiliations:** Monash Alfred Psychiatry Research Centre, Monash University, Central Clinical School and the Alfred Hospital, Melbourne, Australia

**Keywords:** Peak saccadic eye velocity, Menstrual cycle, Allopregnanolone, GABA_A_, Premenstrual dysphoric disorder

## Abstract

Peak saccadic eye velocity (pSEV) has been investigated in studies that characterise the pathophysiology of menstrual cycle related mood disorders, such as premenstrual dysphoric disorder (PMDD). pSEV is a stable and sensitive measure of gamma-aminobutyric acid A (GABA_A_) receptor function. Dysregulation of the GABA pathway has been associated with the onset of PMDD. Despite a growing number of studies utilising pSEV as an outcome measure in interventional drug studies for menstrual cycle related mood disorders, there are no reported studies that have investigated whether pSEV is sensitive to hormone fluctuations across the natural menstrual cycle. To address this gap, this pilot study aimed to characterise pSEV in women across the menstrual cycle. Participants were monitored across two menstrual cycles and saccadic eye movements were measured in both luteal and follicular phases. Seven participants completed the full study and were included in the final analysis. Results revealed luteal phase pSEV was significantly less than follicular phase pSEV. This finding is novel and forms a stepping-stone for further understanding the associations between menstrual hormone profiles and GABA_A_ receptors.

## Highlights

• Peak saccadic eye velocity is used as a proxy measure of GABA_A_ function.
• Peak saccadic eye velocity was measured in the follicular and luteal phases of the menstrual cycle.
• Median peak saccadic eye velocity was significantly lower in luteal phase.
• Saccadic eye velocity is sensitive to hormone fluctuations across menstrual cycle.

## 1 Introduction

Eye movement research has been utilised as a tool to investigate psychiatric disorders for over a century [1]. Eye movement paradigms, particularly saccadic paradigms, assessing rapid shifts of gaze, are useful and sensitive tools to understand neurophysiological mechanisms involved in psychiatric disorders. Within the field of saccade research, manipulation of the behavioural parameters of the task can change the task demands and numerous outcome variables can be analysed, including latency, gain (or accuracy), error-rate (reflecting lack of inhibitory control), and saccadic-eye-velocity [2]. Research investigating many psychiatric disorders has utilised these saccade variables, with well-established saccade findings in schizophrenia and related disorders [1]. Peak saccadic-eye-velocity (pSEV), defined as the greatest speed in a particular direction during a saccade, has gained momentum as an index of drug sensitivity in menstrual cycle related mood disorders, including premenstrual dysphoric disorder (PMDD). PMDD is characterised by dysphoria, anxiety, mood lability and irritability, often also suicidality [3]. PMDD is thought to affect 3–8% of menstruating women and many women do not respond to conventional pharmacological therapy [3].

The aetiology of PMDD is an area of active investigation. There is evidence women with PMDD have an altered sensitivity to the progesterone metabolite allopregnanolone, a positive modulator acting at the GABA_A_ receptor [4]. Such receptor sensitivity has been suggested as a contributing factor, at least in part, to the observed symptoms of the disorder [5].

A link between the GABA pathway and saccadic eye movements was initially reported by Hikosaka and Wurtz in 1985. They reported that with injection of muscimol (GABA_A_ receptor agonist) into the superior colliculus of monkeys, there was a significant reduction of pSEV, whereas bicuculline (GABA_A_ receptor antagonist) injection resulted in unsuppressed saccades with reduced latency [6]. Saccadic parameters have since been investigated further in human subjects and characterised in relation to their specificity to GABA modulation. Consequently, pSEV has been identified as a stable and sensitive measure of GABA_A_ receptor function, and is indirectly proportional to positive stimulation of GABA_A_ receptors [4]. pSEV has been used as a proxy measure of GABA_A_ receptor function in menstrual cycle related mood disorder drug studies that have revealed women with premenstrual mood symptoms are less sensitive to the effects of benzodiazepines and allopregnanolone, positive allosteric modulators of GABA_A_ receptors, as compared to women who do not experience premenstrual mood symptoms [7]. The group differences observed in these studies are limited to the luteal phase, with no differential response observed between groups in the follicular phase [7]. These findings are supported by Nyberg et al. [8] who demonstrated a significant difference in pSEV responses to administration of ethanol between women with PMDD and healthy controls during the luteal phase. Despite a growing number of studies utilising pSEV as an outcome measure in PMDD drug studies, there are no reported studies that have investigated whether pSEV is sensitive to hormone fluctuations across the natural menstrual cycle, in women with or without PMDD. To address this gap, the aim of the current observational study was to determine whether pSEV is sensitive to hormone fluctuations across the natural menstrual cycle in women without PMDD. It was hypothesised that pSEV would be lower in the luteal phase as compared to the follicular phase, corresponding to the natural rise in allopregnanolone during the luteal phase [15].

## 2 Methods

This sub-study was part of a larger project, *Hormones and the Mind,* conducted at the Monash Alfred Psychiatry Research Centre (MAPrc), under ethics approval from the Alfred Ethics Committee and the Monash University Human Research Ethics committee.

Inclusion criteria were that participants were females, aged 18–40 years and experienced regular menstrual cycles. Regular menstrual cycles were defined as being predictable by the participant, or with a regular cycle length spanning 23–40 days, or regulated by hormonal intrauterine device (IUD). Exclusion criteria included experiences of previous head injury, current neurological disorder, pituitary or thyroid abnormalities, ever experiencing psychotic symptoms, history of schizophrenia or bipolar disorder 1, currently pregnant or lactating, currently experiencing substance abuse or dependence and taking hormonal contraception causing anovulation.

Sixteen participants commenced the study. Of those, five were lost to follow-up, one withdrew consent due to personal circumstances and three were unable to complete eye-tracking tasks due to poor visual acuity. Seven participants completed all assessments and were included in the final analysis. Following provision of written informed consent, a screening assessment involved the collection of demographic data, Test of Premorbid Function (TOPF) [9] to estimate premorbid intellectual functioning, completion of the Mini-International Neuropsychiatric Interview (MINI) screen [10] and a brief medical history. First Response™ ovulation testing kits were provided to all participants. This test is a urinary luteinising hormone (LH) test that shows a positive result when there is a LH surge indicating ovulation. Follicular phase was determined as 7 ​± ​2 days following onset of menses. Luteal phase was determined as 10 ​± ​2 days following ovulation. Two assessments corresponding to the follicular and luteal phase were conducted, in addition to participants being asked to track psychological, physical and functional symptoms using the Daily Record of Severity of Problems across two menstrual cycles [11]. The data collected from this was analysed using the Carolina Premenstrual Assessment Scoring System [11] to assess women for any forms of menstrual cycle related mood disorder.

Eye-tracking experiments were completed once in the follicular phase and once in the luteal phase, with the order of assessments counterbalanced between participants. The task was conducted in a quiet dark room. The participant was seated 640 ​mm away from a LED monitor with measurements of 1024 ​× ​768 resolution, 303 ​mm ​× ​378 ​mm. Gaze height was in line with the centre of the screen and the head was stabilised with a chin rest. The EyeLink Portable Duo (SR Research Ltd.) was used, which samples at a rate of 2000 ​Hz. For each participant, the eye tracker was calibrated using a five-point calibration. Drift correction was then performed throughout the task when necessary. A central fixation target, white open circle, was presented for 1500 ​ms. This was immediately followed by a peripheral target (green cross subtending one-degree visual angle) presented at either ​± ​5°, ± 7.5° or ​± ​10° for 1500 ​ms on a black background. The task was designed using the Experiment Builder software provided by SR Research Ltd. The task was blocked and interleaved into pro and antisaccade blocks of 12 trials. Each block was preceded by instruction ‘TOWARD’ or ‘AWAY’ indicating directional nature of the saccades being measured. The ‘TOWARD’ condition required the participant to make a prosaccade and look directly toward the target cross as soon as it appeared. The ‘AWAY’ condition required the participant to make an antisaccade; that is, to NOT look at the target cross, and instead look in the mirror location (i.e. opposite direction, same distance from centre) as soon as the target appeared. Two slowed practice trials were included, and the experimenter provided additional verbal instructions to ensure the participant understood the task.

Eye-tracking data were analysed with EyeLink® ​Data Viewer, and custom-made programs in Microsoft Excel. A saccade analysis report was produced for each experiment from Data Viewer and saccades were defined using the system’s saccade detection algorithm: a saccade velocity threshold of 30°/sec, an acceleration threshold of 8000°/sec^2^ and a motion threshold of 0.15°. For each trial, the first valid saccade (primary saccade) was identified, then further defined as saccade amplitude ≥ 2° and latency ≥ 120 ​msec. Mean values and standard deviations were calculated for peak velocity, latency and error rate. Error rate was defined as the percentage of primary saccades that were generated in the wrong direction, i.e. inhibitory errors. Latency in the antisaccade task was for correct antisaccades. Upper and lower limits were determined as two standard deviations either side of mean. Outliers were then excluded on the basis of these limits.

## 3 Statistical analysis

**Fig. 1:**
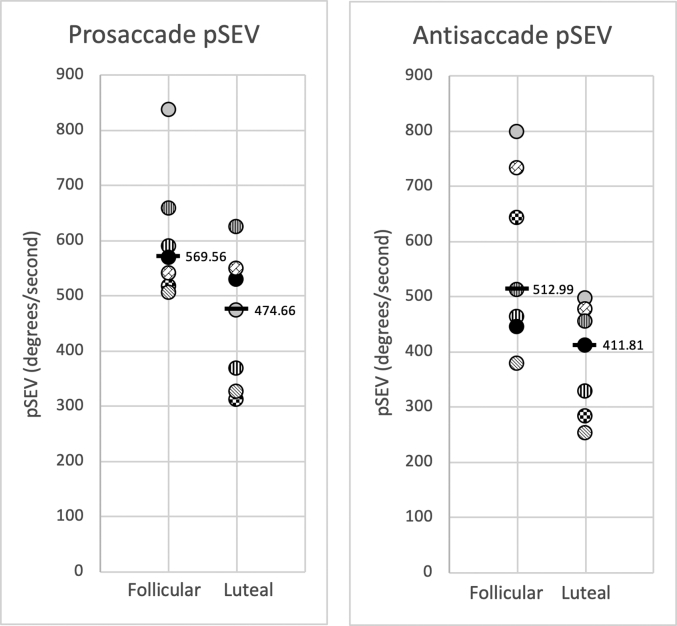
Mean pSEV of each participant, pattern matched, across phases and both pro and antisaccade paradigms. Group medians are represented by bolded horizontal markers, the two sets of black and white vertically striped data points represent the two participants with Mirena® ​IUD, the filled in black data point represents the participant with current major depression and suicidality, the grey filled data point represents the participant with suicidality.

We adopted two approaches for analysis – descriptive (see Table 1) and inferential (Fig. 1). Variables descriptive analyses with means (standard deviations), medians (interquartile range), and range were summarised in Table 1.

**Table 1:** Descriptive data related to age, menstruation, TOPF, pSEV, latency and error rate.

	Mean (STD)	Median (IQR)	Range
Age (years)	24.57 (4.08)	22.00 (22–26)	22–33
Average Length of Cycle (days)	29.57 (5.91)	28.00 (24–33)	23–40
Age of Menarche (years)	12.14 (1.35)	12.00 (11–13)	10–14
TOPF Score (standardised)	120.29 (6.13)	122.00 (115–127)	111–127
PROSACCADE			
pSEV – Follicular (degrees/second)	602.42 (115.18)	569.56 (517.33–657.44)	504.92–836.61
pSEV – Luteal (degrees/second)	454.65 (121.52)	474.66 (326.09–549.15)	311.14–625.20
Latency – Follicular (milliseconds)	183.53 (18.74)	176.44 (166.34–205.00)	162.70–211.04
Latency – Luteal (milliseconds)	186.36 (20.55)	186.47 (170.08–195.86)	160.30–223.67
Percentage Error – Follicular	1.83 (2.63)	0 (0–3.03)	0–6.90
Percentage Error – Luteal	1.31 (2.44)	0 (0–2.94)	0–6.25
ANTISACCADE			
pSEV – Follicular (degrees/second)	567.89 (158.90)	512.99 (445.64–733.96)	377.87–798.35
pSEV – Luteal (degrees/second)	386.45 (97.96)	411.81 (283.59–476.42)	253.06–497.40
Latency – Follicular (milliseconds)	294.82 (70.89)	264.48 (240.75–371.11)	228.20–411.20
Latency – Luteal (milliseconds)	308.51 (63.45)	281.71 (268.15–374.07)	230.39–406.17
Percentage Error – Follicular	37.24 (27.95)	29.17 (14.29–59.26)	6.67–82.76
Percentage Error – Luteal	38.13 (26.02)	42.42 (12.12–57.14)	0–74.07

A parameter-free analysis for correlated data was used as outcome scores were not normally distributed. For the inferential statistics, we conducted two related samples Wilcoxon sign-rank test to test the null hypothesis that there would be no difference between the pSEV between the luteal and follicular phases during two tasks – i) the prosaccade and ii) the antisaccade task. The alpha level was set at two tailed 5%. Analyses were performed with SPSS version 26 (Chicago, IL).

## 4 Results

All participants were right-handed, with no previous pregnancies and no participant met criteria for PMDD. Two participants were using Mirena® IUD but were not excluded as urinary ovulatory kits confirmed ovulatory LH rise, and their results were in keeping with other participants (see Fig. 1). Two participants experienced suicidality at the time of the study, of those two, one ​had current major depression, panic disorder and generalised anxiety disorder as well. This was confirmed using the MINI screen. ​Neither of these two participants showed any deviation from the collective results, demonstrated in Fig. 1. Follicular phase testing occurred on days 4–8 of the menstrual cycle, and luteal phase testing occurred 8–12 days following LH rise.

Table 1 shows the summary statistics for each outcome variable. The results of each Wilcoxon sign-rank hypothesis testing yielded an asymptotic p value of 0.028 for the prosaccade pSEV and 0.018 for the antisaccade pSEV. The p values reveal there is evidence against the null hypothesis, of equal score medians between the follicular and luteal phases. The observed data in Fig. 1 shows the median pSEV is lower in the luteal phase across both pro and antisaccade paradigms.

Table 1 provides demographic data along with values related to pSEV, latency and error rate.

## 5 Discussion

This pilot study examined pSEV during a prosaccade and antisaccade task in naturally cycling females across two menstrual cycle phases – follicular and luteal. Our results found significant phase differences in pSEV in both pro and antisaccade paradigms, whereby the median luteal phase pSEV was significantly lower than the median follicular phase pSEV. No phase differences were observed in other measures of saccade performance, including latency and error rate.

The mechanism hypothesised to underpin menstrual cycle governed changes in pSEV is increased progesterone with consequential rises in allopregnanolone in the luteal phase [15]. This rise leads to positive modulation of GABA_A_ receptors, thought to contribute to a reduction in pSEV [12]. This notion is supported by previous literature which has shown that in healthy menstrual cycles, exogenous administration of positive GABA_A_ receptor modulators results in reduced pSEV [8,13]. Of note, previous studies [[5], [8]] have analysed saccades of larger amplitudes (i.e. 30°) which reflects the maximum saccade velocity reached. Our results demonstrate pSEV differences at smaller amplitudes, it would of interest to expand menstrual cycle phase differences at larger amplitudes in the future. This is the first non-interventional study to investigate pSEV across natural menstrual cycles.

These pilot findings should be interpreted within the context of the study’s strengths and limitations. Strengths include the longitudinal within-subject study design and gold-standard tools employed in assessing menstrual cycle symptoms. The obvious limitation is the small sample size, noting, however, that appropriate statistical analysis was used. A second limitation is the absence of hormone testing.

Despite the pilot nature of this study, clinical implications of this study include support for further use of pSEV as a tool for understanding GABA related changes, particularly in relation to the menstrual cycle. Our knowledge of functional GABA changes within in vivo models is limited and lacks non-invasive research tools [14]. Future research can expand the use of pSEV as a non-invasive tool to investigate the pathophysiology of menstrual cycle related mood disorders such as PMDD. This is a pertinent area for further research, as despite the debilitating impacts PMDD has on affected women, the response to current treatments is low (<60%) [3] and as such there is much to be understood of these conditions to better diagnose and manage them.

In summary, this research is novel in that it investigates the functional GABA changes through measures of pSEV, across the natural menstrual cycle. This research forms a preliminary foundation for future research into pSEV, GABA_A_ receptor function and the menstrual cycle, as well as menstrual cycle related mood disorders.

## Declaration of competing interest

The authors declare that they have no known competing financial interests or personal relationships that could have appeared to influence the work reported in this paper.
